# Integrated analysis of exosomal lncRNA and mRNA expression profiles reveals the involvement of lnc‐MKRN2‐42:1 in the pathogenesis of Parkinson's disease

**DOI:** 10.1111/cns.13277

**Published:** 2019-12-08

**Authors:** Qiao Wang, Chun‐Lei Han, Kai‐Liang Wang, Yun‐Peng Sui, Zhi‐Bao Li, Ning Chen, Shi‐Ying Fan, Michitomo Shimabukuro, Feng Wang, Fan‐Gang Meng

**Affiliations:** ^1^ Department of Functional Neurosurgery Beijing Neurosurgical Institute Capital Medical University Beijing China; ^2^ Beijing Key Laboratory of Neurostimulation Beijing China; ^3^ Department of Neurosurgery Beijing Tiantan Hospital Capital Medical University Beijing China; ^4^ Department of Neurosurgery General Hospital of Ningxia Medical University Yinchuan China

**Keywords:** exosome, Lnc‐MKRN2‐42:1, MDS‐UPDRS III, next‐generation sequencing, Parkinson's disease

## Abstract

**Background:**

Parkinson's disease (PD) is a common movement disorder for which diagnosis mainly depends on the medical history and clinical symptoms. Exosomes are now considered an additional mechanism for intercellular communication, allowing cells to exchange proteins, lipids, and genetic material. Long noncoding (lnc) RNA in exosomes plays a critical role in many diseases, including neurodegenerative disease.

**Aim:**

To study expression differences for lncRNAs in peripheral blood exosomes of PD patients compared with healthy individuals and to look for lncRNAs that might be related to the pathogenesis of PD.

**Materials and Methods:**

We recruited PD patients along with age‐ and sex‐matched healthy individuals as healthy controls and evaluated levels of lncRNAs extracted from exosomes in plasma samples via next‐generation sequencing and real‐time quantitative PCR. Correlation analysis was conducted for the clinical characteristics of PD patients and the expression of selected lncRNAs.

**Results:**

We found 15 upregulated and 24 downregulated exosomal lncRNAs in the PD group. According to bioinformatics analyses, we chose lnc‐MKRN2‐42:1 for further study. Interestingly, lnc‐MKRN2‐42:1 was positively correlated with the MDS‐UPDRS III score for PD patients.

**Conclusion:**

Our study suggested that lnc‐MKRN2‐42:1 may be involved in the occurrence and development of PD.

## 1 INTRODUCTION

Parkinson's disease (PD) is the second most common neurodegenerative disease after Alzheimer's disease, with median age‐standardized annual incidence rates in high‐income countries of 14 per 100 000 individuals in the total population, and 160 per 100 000 among those aged 65 years or older.^1^ The clinical features of PD include resting tremor, rigidity, bradykinesia, and gesture failure, with a higher risk of nonmotor symptoms, including cognitive impairment and dementia.^2^, ^3^ The main pathological changes in PD are the degeneration and death of dopaminergic neurons in the midbrain substantia nigra and significant decreases in dopamine in the striatum, resulting in an imbalance between dopamine and acetylcholine transmitters. The presence of Lewy bodies in substantia nigra cells is considered a pathological marker of PD. However, the exact mechanism underlying the PD remains unclear.^4^ Diagnosis of PD mainly depends on the medical history and clinical symptoms, which are easy to misdiagnose.^3^

Exosomes are cell‐derived membranous structures that originate from the endosomal system. They are present in blood, semen, saliva, urine, amniotic fluid, cerebrospinal, bile, breast milk, and culture medium from cell cultures, and range in size from 30 to 100 nm.^5^ Exosomes are now considered an additional mechanism for intercellular communication. Exosomes contain proteins, RNA, and DNA of their cell of origin, and the lipid bilayer can protect these specific contents from enzymatic degradation. In 2016, Fraser et al demonstrated that levels of autophosphorylated Ser(P)‐1292 LRRK2 are elevated in urinary exosomes in cases of idiopathic PD. They also found that cognitive impairment severity and difficulty in performing daily activities were correlated with levels of urinary exosome Ser(P)‐1292 LRRK2.^6^ Studies have shown that exosomes are involved in the transmission and release of α‐synuclein, which is closely related to PD pathogenesis and dysfunction, and is the main component of Lewy bodies.^7^, ^8^ Given the difficulty in obtaining brain tissue and the properties of exosomes, it is appropriate to use exosomes rather than brain tissue to study PD.

Long noncoding RNAs (lncRNAs) are noncoding RNAs with a length greater than 200 nucleotides (nt) that can comprehensively regulate gene expression through a variety of different mechanisms at epigenetic, transcription, posttranscription, and translation levels.^9^, ^10^ In 2014, Soreq et al used whole‐transcriptome RNA sequencing to screen 13 differentially expressed lncRNAs in peripheral PD leukocytes, five of which contained the spliceosome component U1, which supported the idea that splicing modulations were involved in the disease. Lnc‐fr91.3 was believed to be related to muscle rigidity in PD.^11^ In 2015, Carrieri et al demonstrated that antisense transcription regulated sense genes functioned at distinct regulatory levels in PD and could be used as a new therapeutic strategy.^12^ In 2016, Kraus et al analyzed the lncRNA expression profile of brain tissues from PD patients and normal subjects and found that significant differences in the expression of lncRNA‐p21, MALAT1, SNHG1, NEAT1, and H19. Such differences appeared in the early stages of PD and accompanied its development.^13^ These studies suggested that lncRNAs play an important role in the pathogenesis of PD, but the expression of lncRNAs in peripheral blood exosomes of PD patients remains unknown. In this study, we screened lncRNAs in peripheral exosomes from PD patients in order to identify valuable lncRNAs for further study.

## 2 METHOD

### 2.1 Participants

Thirty‐two PD patients and 13 healthy volunteers from Beijing Tiantan Hospital signed informed consent and agreed to undergo evaluations that consisted of a medical history, physical examination (including neurological), laboratory tests, and neuropsychological assessments. Laboratory evaluations included a complete blood count, serum electrolytes, blood urea nitrogen, and creatinine. All results were within normal limits. The local medical ethics committee approved this study (Beijing Tiantan Hospital, Capital Medical University, Beijing, China; reference number KYSQ 2019‐103‐01). Demographic information for all subjects and patients is listed in Table 1.

**Table 1:** Overview of samples

Case	Diagnosis	Sex	Age (y)	Course (y)	MDS‐UPDRS3	MoCA	HAMA	HAMD
Dnr_01	PD	Male	52	6	52	9	28	30
Dnr_02	PD	Male	53	7	56			
Dnr_03	PD	Male	72	8	67			
Dnr_04	PD	Female	70	10	53	24	10	10
Dnr_05	PD	Female	54	16	71	18	45	44
Dnr_06	PD	Male	55	6	53	20	6	12
Dnr_07	PD	Female	74	5	52	17	29	30
Dnr_08	PD	Female	64	6	56	26	6	12
Dnr_09	PD	Male	76	21	88.6	16	31	17
Dnr_10	PD	Male	76	14	81	26	20	18
Dnr_11	PD	Male	66	12	73.1	26	14	5
Dnr_12	PD	Male	74	4	52.6	18	26	20
Dnr_13	PD	Male	64	8	69.4	22	12	13
Dnr_14	PD	Male	57	10	83	22	17	9
Dnr_15	PD	Male	68	9	73	26	21	16
Dnr_16	PD	Female	74	10	62	21	12	11
Dnr_17	PD	Male	63	5	46.7	20	22	30
Dnr_18	PD	Female	54	8	77.8	23	12	14
Dnr_19	PD	Male	66	10	61	24	10	2
Dnr_20	PD	Male	65	4	35.9	22	15	11
Dnr_21	PD	Male	59	8	63	24	15	6
Dnr_22	PD	Female	64	3	47	25	21	22
Dnr_23	PD	Female	50	5	52	18	17	10
Dnr_24	PD	Male	50	7	77	24	13	10
Dnr_25	PD	Female	66	14	18	14	14	8
Dnr_26	PD	Male	43	8	48	24	32	21
Dnr_27	PD	Female	54	10	37	22	11	21
Dnr_28	PD	Female	69	10	75	26	36	26
Dnr_29	PD	Female	38	10	63	22	15	16
Dnr_30	PD	Male	60	6	54	17	15	14
Dnr_31	PD	Male	64	14	34	15	17	13
Dnr_32	PD	Female	68	9	61			
Dnr_33	HC	Male	25					
Dnr_34	HC	Male	53					
Dnr_35	HC	Male	51					
Dnr_36	HC	Male	49					
Dnr_37	HC	Male	60					
Dnr_38	HC	Female	62					
Dnr_39	HC	Female	56					
Dnr_40	HC	Female	57					
Dnr_41	HC	Female	52					
Dnr_42	HC	Female	49					
Dnr_43	HC	Male	45					
Dnr_44	HC	Male	60					
Dnr_45	HC	Male	67					

Abbreviations: HAMA, Hamilton Anxiety Scale; HAMD, Hamilton Depression Scale; HC, healthy control; MDS‐UPDRS, Movement Disorder Society‐Sponsored Revision Unified Parkinson's Disease Rating Scale; MoCA, Montreal Cognitive Assessment; PD, Parkinson's disease.

### 2.2 Exosome collection

Seven PD patients (Dnr_01 to Dnr_07; age 63 ± 11 years) and seven healthy controls (Dnr_34 to Dnr_40; age 58 ± 9 years) were selected for next‐generation sequencing (NGS) of exosome lncRNAs. There was no significant difference in age between the two groups, and each group consisted of four males and three females. Peripheral blood samples from individuals were collected in ethylene diamine tetraacetic acid (EDTA) tubes following a regular venipuncture procedure. After centrifugation at 3000 × *g* for 15 minutes at 4°C, the plasma was aspirated and stored at −80°C before use. The ultracentrifugation method was optimized according to the method previously described.^14^ After thawing at 37°C, plasma samples were centrifuged at 3,00 × *g* for 15 minutes to remove cell debris. The supernatant was then diluted using a sevenfold volume of phosphate‐buffered saline (PBS), centrifuged at 13 000 × *g* for 30 minutes, and processed through a 0.22 μm filter to remove large particles. The supernatant was ultracentrifuged using a P50A72‐986 rotor (CP100NX; Hitachi, Brea, CA, USA) at 100 000 × *g* at 4°C for 2 hours to pellet the exosomes. The pellet was resuspended in PBS and centrifuged again at 100 000 × *g* at 4°C for 2 hours. After PBS washing, the exosome pellet was resuspended in 100 µL of PBS.

### 2.3 Exosome identification

Exosomes were identified via TEM, NTA, and Western blotting. A 20‐µL aliquot of exosome solution was placed on a copper mesh and incubated at room temperature for 10 minutes. After washing with sterile distilled water, the exosomes were contrasted with uranyl oxalate solution for 1 minute. The sample was then dried for 2 minutes under incandescent light. The copper mesh was observed and photographed using a transmission electron microscope (JEOL‐JEM1400, Tokyo, Japan). Vesicle suspensions with concentrations between 1 × 10^7^/mL and 1 × 10^9^/mL were examined using a ZetaView PMX 110 instrument (Particle Metrix, Meerbusch, Germany) equipped with a 405‐nm laser to determine the size and quantity of particles isolated. A video of 60‐s duration was recorded at a frame rate of 30 frames/s, and particle movement was analyzed using NTA software (ZetaView 8.02.28). The exosome supernatants were denatured in 5 × sodium dodecyl sulfate (SDS) buffer and subjected to Western blot analysis (10% SDS‐polyacrylamide gel electrophoresis; 50 µg protein/lane) using rabbit polyclonal antibody CD63 (sc‐5275; Santa Cruz Biotechnology, Santa Cruz, CA, USA), TSG101 (sc‐13611; Santa Cruz Biotechnology), and calnexin (10427‐2‐AP; Promega, Madison, WI, USA). The antibody dilutions used for Western blots were 1:200 for TSG101 and CD63, and 1:1000 for Calnexin. The proteins were visualized on a Tanon 4600 automatic chemiluminescence image analysis system (Tanon, Shanghai, China).

### 2.4 ExoRNA isolation and RNA analyses

Total RNA was extracted and purified from plasma exosomes using a miRNeasy^®^ Mini kit (Qiagen, Redwood City, CA, USA; cat. #217004) according to the kit instructions. RNA degradation and contamination, especially DNA contamination, were monitored on 1.5% agarose gels. The RNA concentration and purity were evaluated using a NanoDrop 2000 spectrophotometer (Thermo Fisher Scientific, Wilmington, DE, USA). RNA integrity was assessed using an RNA Nano 6000 assay kit on an Agilent Bioanalyzer 2100 system (Agilent Technologies, Santa Clara, CA, USA).

A total of 5 ng of RNA per sample was used as input material for rRNA removal using a Ribo‐Zero™ Magnetic kit (Epicentre, Madison, WI, USA). Sequencing libraries were generated using an Ovation RNA‐Seq system (NuGEN, Redwood City, CA, USA) following the manufacturer's recommendations, and index codes were added to attribute sequences to each sample. For small RNA libraries, a total amount of 2.5 μg of RNA per sample was used as input material for the RNA sample preparation. Sequencing libraries were generated using an NEB Next Multiplex Small RNA Library Prep Set for an Illumina kit (New England Biolabs, Ipswich, MA, USA) following the manufacturer's recommendations. Index codes were added to attribute sequences to each sample. Finally, the PCR products were purified (AMPure XP system; Beckman Coulter, Brea, CA, USA) and library quality was assessed on an Agilent Bioanalyzer 2100 and by qPCR. Clustering of the index‐coded samples was performed on an acBot cluster generation system using TruSeq PE Cluster Kitv3‐cBot‐HS (Illumina, Foster City, CA, USA) according to the manufacturer's instructions. After cluster generation, the library preparations were sequenced on an Illumina Hiseq platform and paired‐end reads were generated. The transcriptome was assembled using the Cufflinks and Scripture programs based on threads mapped to the reference genome. The assembled transcripts were annotated using the Cuffcompare program from the Cufflinks package. Unknown transcripts were used to screen for putative lncRNAs. Three computational approaches (CPC, CNCI and Pfam) were combined to sort nonprotein‐coding RNA candidates from putative protein‐coding RNAs among the unknown transcripts. Putative protein‐coding RNAs were filtered out using a minimum length and exon number threshold. Transcripts longer than 200 nt and having more than two exons were selected as lncRNA candidates and further screened using CPC/CNCI/Pfam, which has the power to distinguish protein‐coding from noncoding genes. In addition to the different types of lncRNAs, lincRNA, intronic lncRNA, and antisense lncRNAs were selected using Cuffcompare (Figure S6).

Cuffdiff (v2.1.1) was used to calculate the fragments per kilobase of exon per million reads (FPKMs) for both lncRNAs and coding genes in each sample.^15^ Gene FPKMs were computed by summing the FPKMs for transcripts in each gene group. FPKM was calculated based on the length of a fragment and read counts mapped to this fragment. Differential expression analysis of two conditions/groups was performed using the Mann‐Whitney *U* test with significance cutoffs of *P* ≤ .05 and |log2(fold‐change)| ≥ 1.5.

### 2.5 GO and KEGG pathway enrichment analysis

We predicted the target genes for the differentially expressed lncRNAs. Perl script was used to identify adjacent genes in the range of 100kb upstream and downstream of lncRNAs as cis target genes of lncRNAs. LncTar was used to predict the trans target genes of our lncRNAs.^16^ Gene Ontology (GO) enrichment analysis of the target genes for differentially expressed lncRNAs was carried out using the GOseq R packages based on a Wallenius noncentral hyper‐geometric distribution. Kyoto Encyclopedia of Genes and Genomes (KEGG) is a database resource for understanding high‐level functions and utilities of biological system (such as cell, organism, and ecosystem) from molecular‐level information, especially large‐scale molecular datasets generated via genome sequencing and other high‐throughput experimental technologies (http://www.genome.jp/kegg/).^17^ We used KOBAS^18^ to test the statistical enrichment of differentially expressed genes in KEGG pathways.

### 2.6 Quantitative polymerase chain reaction (qPCR)

Total RNA was extracted from plasma samples from 24 PD patients (Dnr_8 to Dnr_31) and 11 healthy controls (Dnr_35 to Dnr_45) using an exoRNeasy Serum/Plasma Maxi Kit (50) (Qiagen, Redwood City, CA, USA; cat. #77064). The DNA in the RNA residue was digested using a DNase I kit (CW Bio, Cambridge, MA, USA; cat. #CW2090). The RNA was reverse transcribed into complementary DNA (cDNA) using a HiFi‐MMLVcDNA kit (CW Bio; cat #CW0744) according to the manufacturer's instructions. PCR was performed on the cDNA using UltraSYBR Mixture (With ROX; CW Bio; cat #CW0956). No electrophoresis was performed because of a low RNA yield. β‐actin was used as a housekeeping gene. Data were calculated as relative expressions according to the ^ΔΔ^C(t) principle.^18^, ^19^ The primer sequences were 5′‐GCA AGC CTA ACT CAA GCC ATT‐3′ and 5′‐TCA AGC CGA CTC TCC ATA CC‐3′ for GAS5:46; and 5′‐AGG TGG GAG GAT CGC TTG A‐3′ and 5′‐ACC ATA TTG ATG CCG AAC TTA GTG‐3′ for lnc‐MKRN2‐42:1.

### 2.7 Statistical analysis

Data were analyzed using SPSS version 19.0 statistical software and GraphPad Prism 7.0. Data in all figures are expressed as mean ± standard error of the mean (s.e.m.). Data were compared using Student's *t*‐test (two groups). The Spearman's rank test was used for statistical analysis of the gene expression data set and clinical characteristics. *P* < .05 was considered statistically significant.

## 3 RESULTS

### 3.1 Clinical characteristics and exosome characterization and properties

**Figure 1:**
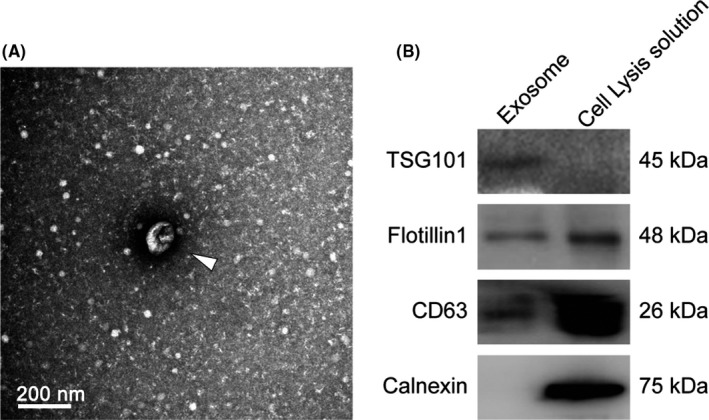
Identification of exosomes. A, Transmission electron microscopy. B, Western blotting. Arrow indicates an exosome in (A). Cell lysis solution is the positive control of the WB experiment

Clinical characteristics were collected from medical records (Table 1). Movement Disorder Society‐Sponsored Revision Unified Parkinson's Disease Rating Scale (MDS‐UPDRS) III, Montreal Cognitive Assessment (MoCA), Hamilton Depression Scale (HAMD), and Hamilton Anxiety Scale (HAMA) scores were all evaluated by two physicians specializing in movement disorders. We used ultracentrifugation to isolate exosomes from human peripheral blood for NGS. Exosomes were verified using transmission electron microscopy (TEM), nanoparticle tracking analysis (NTA), and Western blots. TEM images showed exosomes as small cap‐shaped membrane vesicles after fixation, adhesion, negative staining, and visualization (Figure 1A), consistent with previous observations.^5^ NTA is a method for direct, real‐time visualization and analysis of nanoparticles in liquids and relates the rate of Brownian motion to particle size.^20^ The mean diameter of exosomes observed via NTA was consistent with the TEM results, confirming that the exosomes were small extracellular vesicles.^21^ CD63 and TSG101 were used as exosome surface protein markers, while calnexin was used as a negative marker,^22^ and their presence and absence were verified by our Western blot results (Figure 1B).

### 3.2 Differential expression of lncRNAs and mRNAs between PD and healthy control individuals

**Figure 2:**
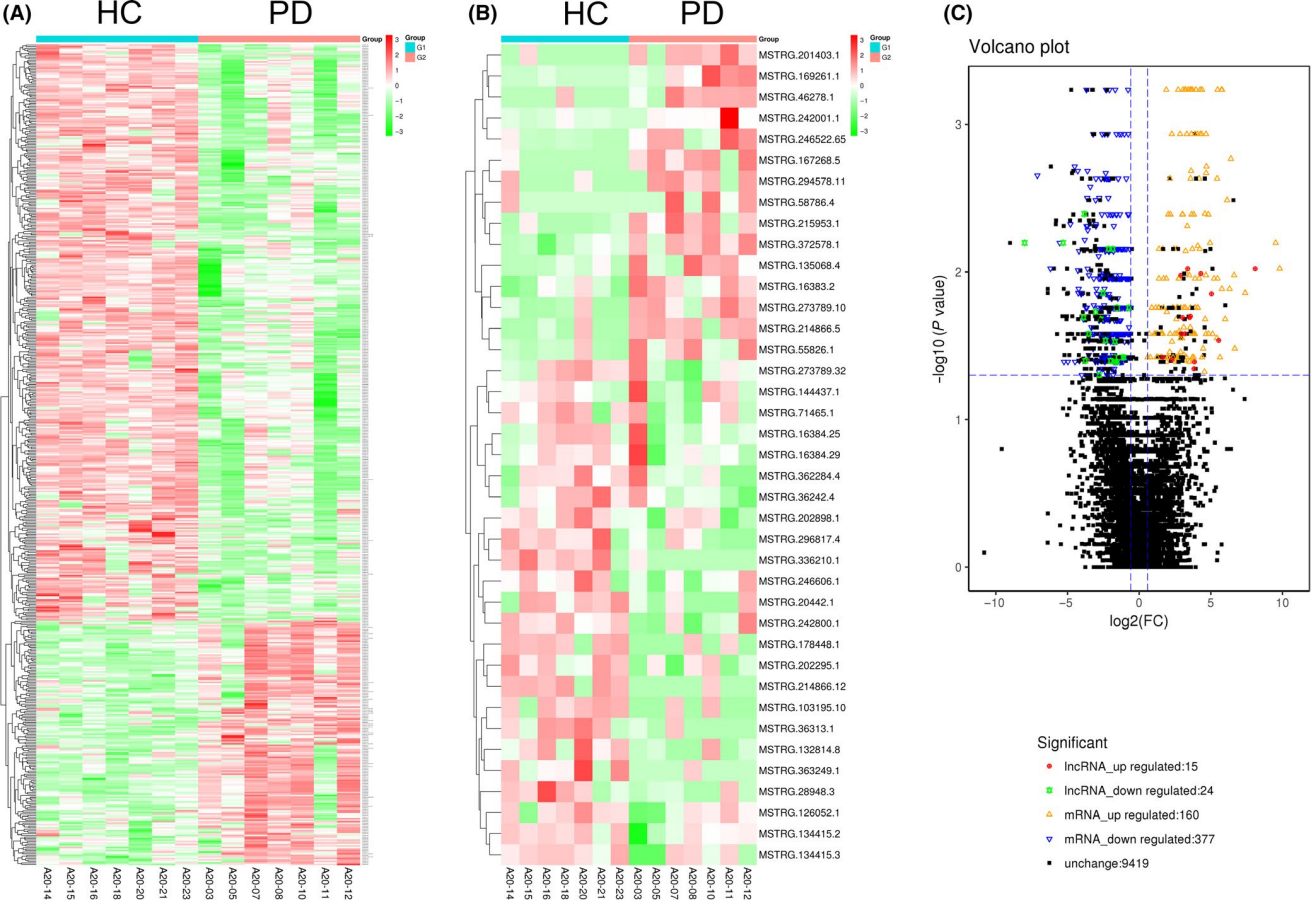
Heatmap of differentially expressed mRNAs (A) and lncRNAs (B) in PD patients comparing with healthy control by sequencing. Volcano Plot of differentially expressed mRNA and lncRNA in PD patients comparing with healthy control by sequencing (C). A, B, Different columns represent different samples (n = 14), and different rows represent different genes. The color bar refers to the log10FPKM of lncRNA. “Red” indicates high relative expression, and “green” indicates low relative expression. PD, Parkinson's disease; HC, healthy control. C, Each point represents a gene, red represents upregulated lncRNA, green represents downregulated lncRNA, orange represents upregulated genes, blue represents downregulated genes, and black represents nondifferentially expressed genes

All lncRNAs obtained from exosomal lncRNAs from PD patients and healthy control subjects were deep sequenced via NGS. The Q30 quality score for each sample was ≥91.54%. FPKM distributions for each sample are shown in (Figure S1). Total clean reads ranged from 124 679 284 to 194 592 044, with an average of 147 178 671. In total, 11.70% to 48.21% sequence reads aligned to the human genome sequence GRCh38. We used |log2(fold‐change)| ≥ 1.5 and *P* ≤ .05 as the thresholds for significantly differential expression. The results revealed 160 upregulated and 377 downregulated mRNAs, 70 upregulated and 41 downregulated miRNAs, 15 upregulated and 24 downregulated lncRNAs, and 62 upregulated and 37 downregulated circRNAs. The most differentially expressed lncRNAs are listed in Table S1. The location of genes on chromosomes is significantly related to their function. Therefore, we analyzed the distribution of differentially expressed lncRNA and mRNA sequences on chromosomes (Figure S2). According to the lncRNA differential expression results, MSTRG.336210.1 and lnc‐MKRN2‐42:1 were highly expressed among healthy subjects, while MSTRG.242001.1 and MSTRG.169261.1 were highly expressed among PD patients. The heatmap in Figure 2A, B provides a visual representation of the differences in mRNA and lncRNA expression levels between the healthy and PD groups. The most differentially expressed transcriptomes are listed in Table S2. ACRBP, CXCL5, ENKUR, and others were highly expressed among PD patients, whereas NME4, CD3D, and ECSCR were highly expressed among healthy subjects.

### 3.3 Functional annotation and enrichment analysis

**Figure 3:**
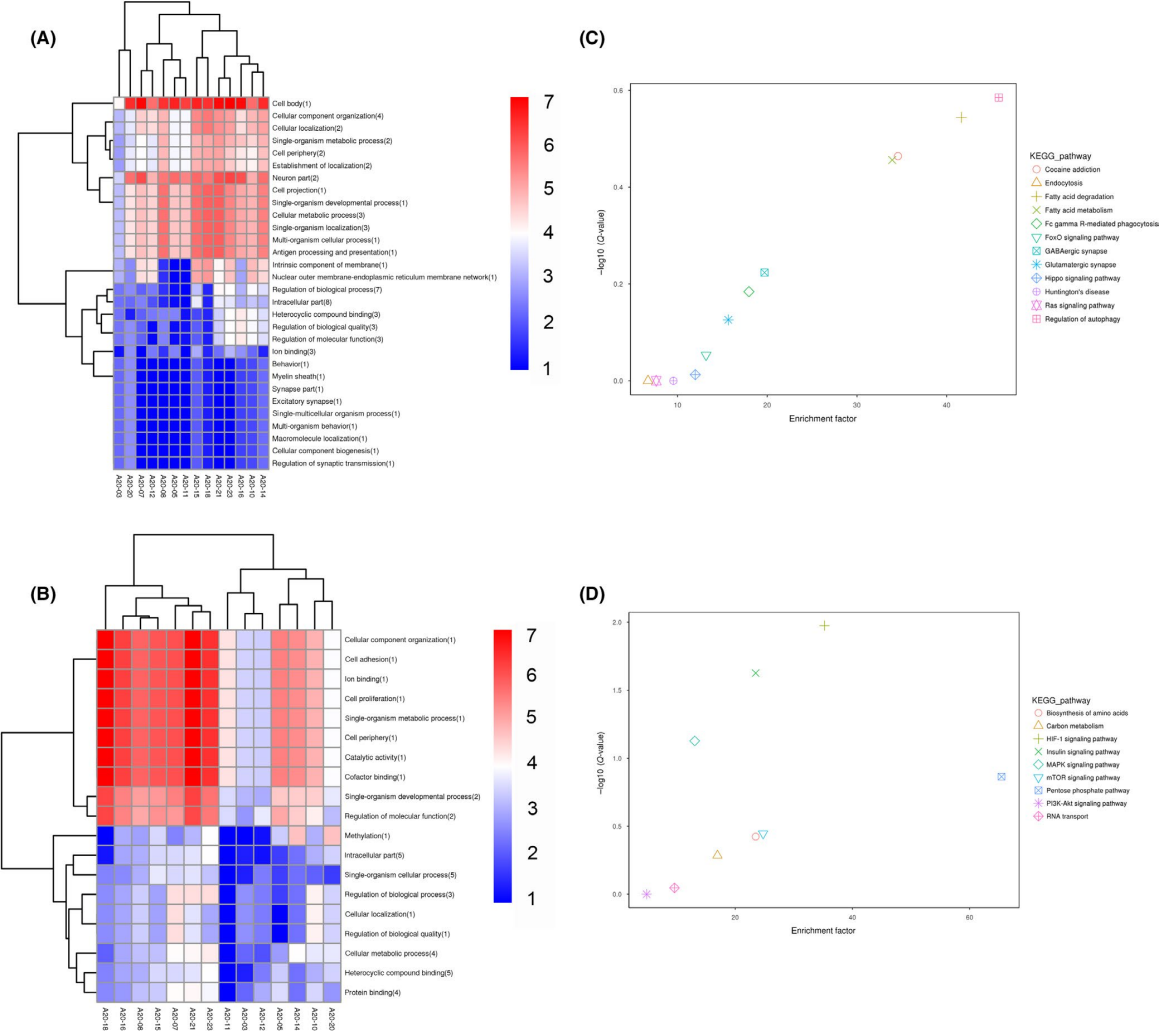
The GO cluster (A, C) and KEGG pathway enrichment analysis (B, D) of target genes of differentially expressed lncRNA. A, C, Red represents high‐expression functional classification, while blue represents metabolic pathways with relatively low expression. B, D, Each figure represents a KEGG pathway. Horizontal axis is enrichment factor. The ordinate is ‐log10 (*q*‐value)

We performed functional annotation of the target genes of differentially expressed lncRNAs. Nine cis target genes and seven trans target genes were functionally annotated. GO analysis showed that these genes are involved in “intracellular part,” “single‐organism cellular process,” “heterocyclic compound binding,” etc (Figure 3) The pathways with the most significant enrichment are listed in Figure 3 (B, D). Remarkably, these target genes were enriched in “autophagy,” “fatty acid degradation,” “pentose phosphate pathway,” and “HIF‐1 signaling pathway.”

To better understand the functions of the differentially expressed mRNAs described above, we performed GO and KEGG pathway enrichment analyses. Go annotations were obtained for approximately 499 differentially expressed mRNAs. GO analysis of these mRNA showed that they are involved in “immune system,” “biological phase,” and “cell killing” biological processes; are primarily enriched in “organelle part” and “macromolecular complex” cellular components; and are involved in molecular functions that include “translation regulator activity,” “structural molecular activity,” and “electron carrier activities” (Figure S3). In addition, KEGG annotation for 369 mRNAs were obtained (Figure S4). The pathways with the most significant enrichment are shown in Figure 3. Remarkably, these mRNAs were enriched in “the ribosome pathway,” “oxidative phosphorylation pathway,” “Parkinson's disease pathway,” and “Huntington's disease pathway” among others.

### 3.4 Target gene prediction for lncRNA and qPCR validation

On the basis of the mode of action of lncRNAs and their target genes, we predicted the target genes for lnc‐MKRN2‐42:1 and GAS5:46. The results for lncRNA and mRNA information analyses suggest that lnc‐MKRN2‐42:1 has a regulatory relationship with multiple target genes (Table 2). The target genes for lnc‐MKRN2‐42:1 are EIF4E, MKNK1, BTD, TMEM78, ZNF428, AC133555.3, ARHGAP8, METTL5, PACRG, GENPL, AC003002.2, ENSG00000279282, ENSG00000280175, etc The target genes for GAS5:46 are CENPL, ZBTB37, SERPINC1, etc

**Table 2:** Target genes of lnc‐MKRN2‐42:1

Target genes	Target type
BTD	cis
AL354828.2	trans
RP11‐345J4.3	trans
ZNF428	trans
MKNK1	trans
TMEM78	trans
AC004076.7	trans
ARHGAP8	trans
EIF4E	trans
RP11‐347C12.3	trans
CENPL	trans
METTL5	trans
AL354828.1	trans
PACRG	trans

**Figure 4:**
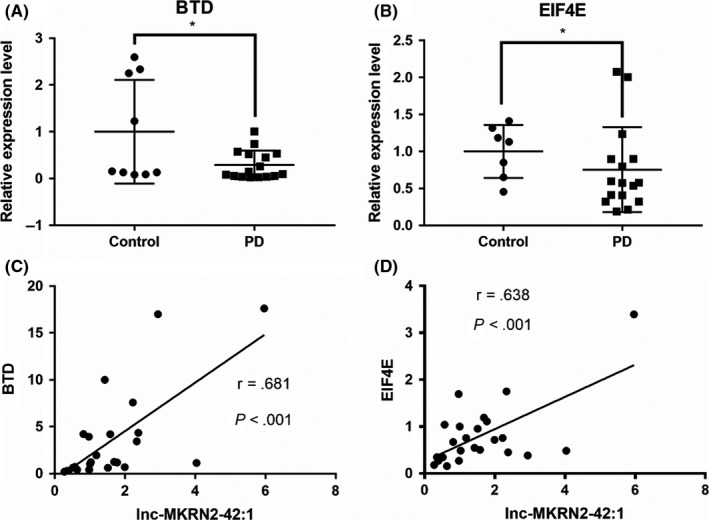
Expression (A, B) and correlation (C, D) of target genes. PD, Parkinson's disease. (A, B) The expression levels of BTD and EIF4E in PD patients as determined using qPCR (n = 11‐24). C, D, Correlations according to spearman coefficient between the related expression level of target gene and the lncRNAs in PD patients. (n = 24)

The downregulation trends for lnc‐MKRN2‐42:1 and GAS5:46 observed via qPCR are consistent with the NGS results. Compared with the healthy control group, BTD expression was approximately 71% lower and EIF4E expression was approximately 25% lower in the PD group, and the expression levels were positively correlated with lnc‐MKRN2‐42:1 (Figure 4). Therefore, we selected lnc‐MKRN2‐42:1 and its target genes for further correlation analysis.

### 3.5 Correlation analysis of differential expression and clinical characteristics

**Figure 5:**
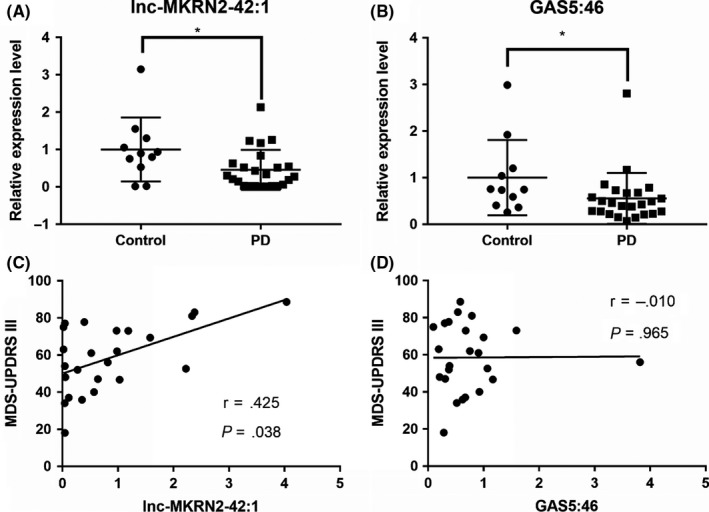
Results of qPCR (A, B) and correlation analysis of lncRNA expression level and clinical characteristics (C, D). PD, Parkinson's disease. MDS‐UPDRS, Movement Disorder Society‐Sponsored Revision Unified Parkinson's Disease Rating. A, B, The expression levels of lnc‐MKRN2‐42:1 and GAS5:46 in PD patients as determined using qPCR (n = 11‐24). C, D, Correlations according to spearman coefficient between the related expression level of lncRNAs and the MDS‐UPDRS III in PD patients. (n = 24)

Correlation analysis was conducted between the clinical characteristics of PD patients and lnc‐MKRN2‐42:1 and GAS5:46 expression. The results demonstrate that lnc‐MKRN2‐42:1 expression was not correlated with HAMA, HAMD, and MoCA scores in PD (Figure S5). However, lnc‐MKRN2‐42:1 expression was positively correlated with MDS‐UPDRS III score among PD patients. GAS5:46 was not correlated with any of the scales (Figure 5).

## 4 DISCUSSION

**Figure 6:**
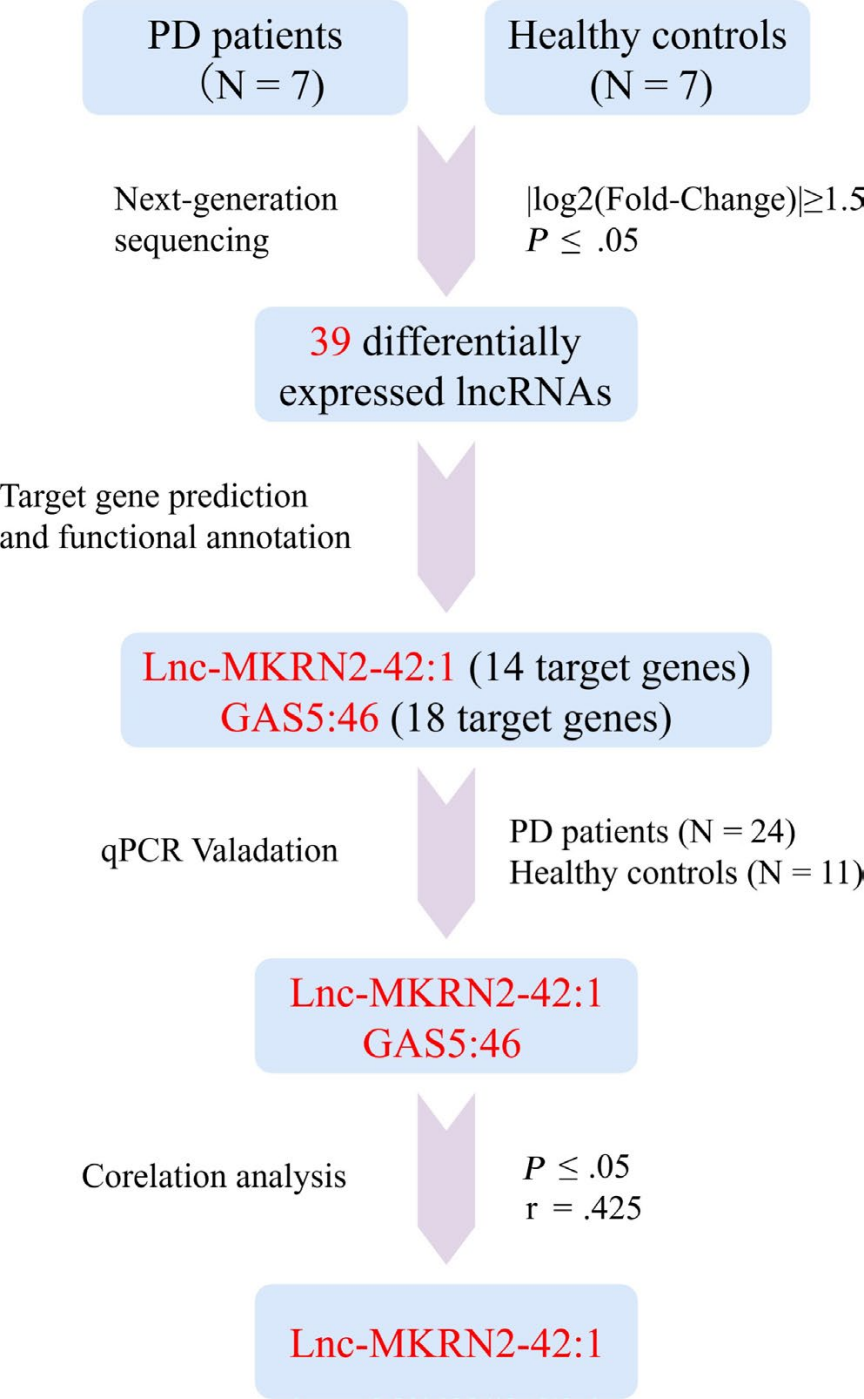
Flowchart of the lnc‐MKRN2‐42:1 selection process

This study included 32 PD patients and 13 healthy controls. We quantitatively analyzed lncRNA expression in peripheral blood vesicles from PD patients and healthy controls using NGS and real‐time quantitative PCR. We identified 15 upregulated and 24 downregulated lncRNAs, of which lnc‐MKRN2‐42:1 was selected for further study. Bioinformatics analyses showed that lnc‐MKRN2‐42:1 could trans‐regulate target genes such as *BTD, EIF4E, MKNK1*, and *METTL5*, involved in biological functions including apoptosis, synaptic remodeling, long‐term potential, immunity, and glutamate neurotransmitter metabolism. Clinical correlation analyses showed that lnc‐MKRN2‐42:1 was positively correlated with MDS‐UPDRS III scores for PD patients, suggesting that this lncRNA may be involved in the occurrence and development of PD. The process used to select lnc‐MKRN2‐42:1 is shown in Figure 6.

To ensure the experimental quality as far as possible, diseases that may cause changes in lncRNA expression levels were strictly excluded, and age and sex ratios were strictly defined in the selection of subjects for sequencing.

Compared with direct detection in peripheral blood, genetic materials in peripheral blood exosomes are more stable and can reflect pathological changes in the central nervous system (CNS). The study of exosomes in disease could provide important clues to the progression of neurodegenerative diseases and cancer. Previous studies provided evidence that amyloids involved in Alzheimer's disease, such as amyloid β, are released via exosomes and that exosome‐associated amyloids can act as seeds for plaque formation.^23^ Release of brain tumor vesicles and uptake by normal cells are associated with progression, angiogenesis, tumor cell invasion, and suppression of immune responses to the tumor. MiR‐21 levels are higher in exosomes from the cerebrospinal fluid of glioblastoma patients when compared to healthy controls, while higher levels of MiR‐21 are associated with poor prognosis and tumor recurrence.^24^ Previous studies also showed that exosomes released by prion‐infected neuronal cells had higher let‐7b, let‐7i, miR‐128a, miR‐21, miR‐222, miR‐29b, miR‐342‐3p, and miR‐424 levels and lower miR‐146a levels when compared to noninfected exosomes.^25^ In the context of immune‐mediated neuroinflammatory diseases, exosomes isolated from interferon‐γ–stimulated dendritic cells, which are potent antigen‐presenting immune cells, were enriched in miRNAs previously associated with anti‐inflammatory processes, including miR‐219.^25^ Brain extracts from a repetitive controlled cortical impact mouse model induced an increase in miR‐124 in cultured BV2 microglia and their associated exosomes.^26^ Shi et al discovered that CNS‐derived exosomes can efflux into blood and that the level of α‐synuclein from CNS‐derived exosomes in plasma is substantially higher in PD patients and is associated with PD severity.^27^ Another study showed that exosomal miR‐331‐5p and miR‐505 have diagnostic value for PD.^28^

Recent studies have shown that lncRNAs are closely related to the pathogenesis of PD. High‐throughput gene chip and sequencing screening results showed that a large number of lncRNAs are differentially expressed in brain tissues 13, 29 and peripheral blood 11 of PD patients, PD animals,^30^ and cell models 31, 32 of PD, suggesting that lncRNAs are involved in the occurrence of PD. Abnormal expression of lncRNAs was observed in the early stages of PD.^33^, ^34^ Preliminary functional studies confirmed that lncRNAs are involved in the occurrence and development of PD. Studies have shown that the lncRNA, MAPT‐AS1, with low expression in PD patients, can increase methylation of the MAPT promoter region and further increase expression of the MAPT gene.^31^ The lncRNA MALAT1 increased the protein stability of α‐synuclein in SH‐SY5Y cells induced by MPP+.^32^ NEAT1, which is highly expressed in PD animals and cell models, can inhibit the degradation of PINK1 protein, and artificially disturbed NEAT1 can inhibit autophagy, improving damage to dopaminergic neurons.^35^ These studies suggest that lncRNAs play important roles in the pathogenesis of PD at an epigenetics level. However, the studies to date have not been systematic or in depth are mostly confined to in vitro analyses, and we know little about the roles of lncRNAs in exosomes in the pathogenesis of sporadic PD.

Lnc‐MKRN2‐42:1 is a newly discovered intergenic antisense lncRNA, located on human chromosome 3. It has a total length of 294 nt and contains only one exon. Currently, there are no reports on lnc‐MKRN2‐42:1. As reported previously, EIF4E is involved in nuclear‐transcribed mRNA catabolic processes, deadenylation‐dependent decay, G1/S transition in the mitotic cell cycle, nuclear‐transcribed mRNA poly(A) tail shortening, and mRNA export from the nucleus.^36^

The clinical manifestations of PD are complex. In addition to the typical movement disorder, balance impairments, restless leg syndrome, depression, anxiety, cognitive disorder, autonomic dysfunction, REM sleep behavior disorder, and speech disorder can occur. Therefore, we used the MDS‐UPDRS, H&Y stage, and Berg Balance Scale to evaluate dyskinesia, HAMA to detect anxiety, HAMD to detect depression, and MMSE and MoCA to detect cognitive impairment.^37^ We analyzed the correlation between the results for most scales and lncRNA expression levels (Figure S5). Other scales were only used to determine the severity of a patient's disease for more careful screening of the enrolled subjects. According to our results, lnc‐MKRN2‐42:1 expression levels were positively correlated with the severity of dyskinesia and dysarthria but were not correlated with other clinical symptoms, so only the correlation analysis for UPDRS III is presented here. Motor dysfunction, including tremor and rigidity, is the most noticeable symptom for PD patients and clinicians, and improvement in motor symptoms is the main index used to evaluate clinical efficacy. Therefore, lnc‐MKRN2‐42:1 could be used as a more objective and stable indicator to judge the development of a patient's condition after further validation in a larger sample.

The sample size in this study is insufficient, and validation in a larger sample is still needed. It must be noted that the plasma samples used in this study were only from clinically diagnosed PD patients and further studies will be necessary to evaluate lncRNA expression in the early stages of PD. Future research will need to investigate whether lnc‐MKRN2‐42:1 has biological functions in PD pathogenesis at the animal and cellular levels.

In conclusion, we discovered that peripheral blood exosomes contained PD‐associated lnc‐MKRN2‐42:1, and its expression level was correlated with PD patients’ clinical scores. Further research will explore whether lnc‐MKRN2‐42:1 is suitable as a new biomarker for PD.

## CONFLICTS OF INTERESTS

The authors have no conflicts of interest to disclose.
