# Supplementary material for: Integrated analysis of exosomal lncRNA and mRNA expression profiles reveals the involvement of lnc‐MKRN2‐42:1 in the pathogenesis of Parkinson's disease
Source: CNS Neurosci Ther. 2019 Dec 8;26(5):527–37. doi: 10.1111/cns.13277 (PMC7163584; doi:10.1111/cns.13277)
Supplement: Supplementary file 1 [file CNS-26-527-s001.docx]

**Figure S1**
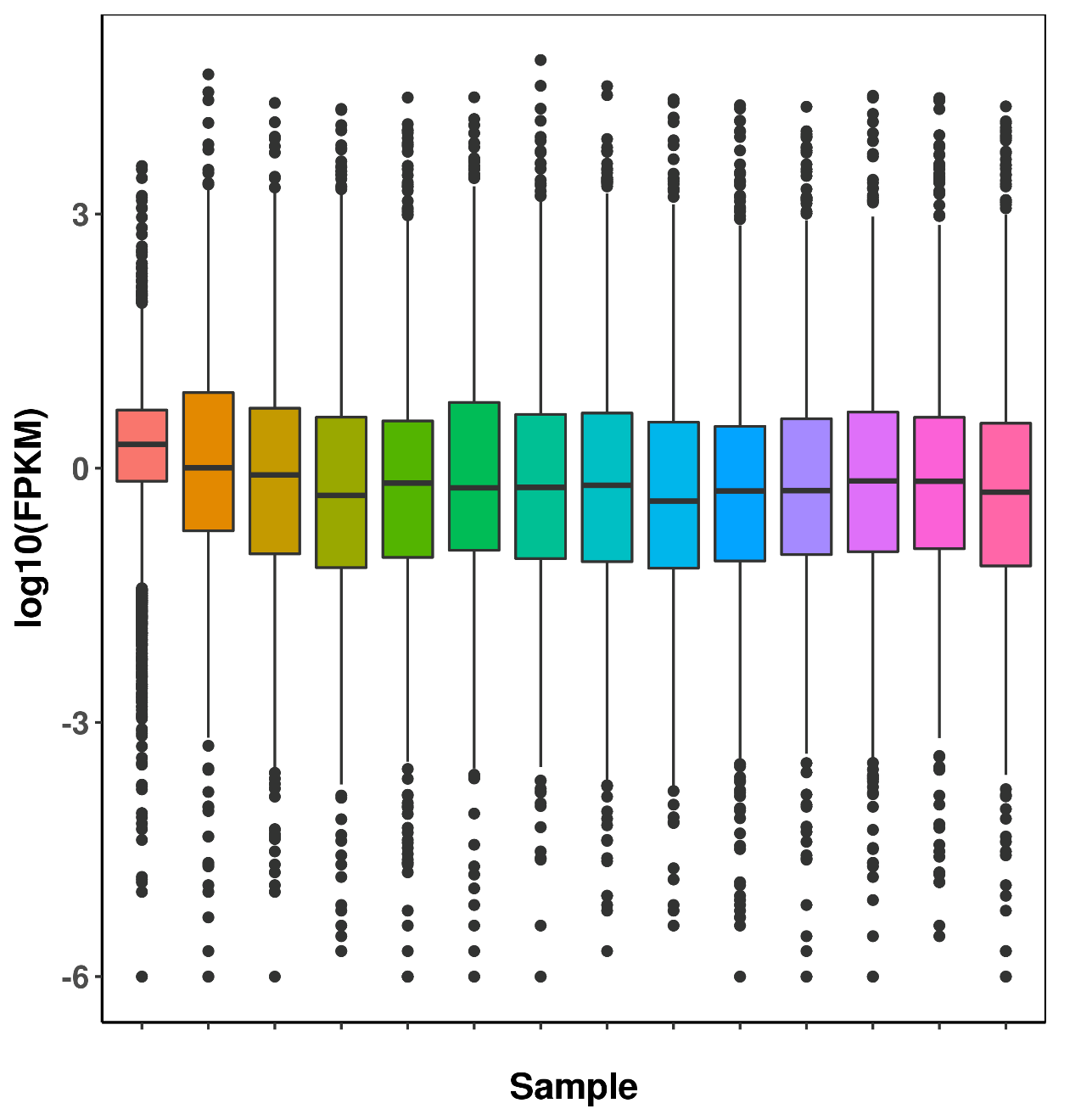
**. Boxplot of FPKM distribution of each sample.** The abscissa represents different samples (n=14); the ordinate represents the logarithm value of sample expression FPKM. This figure measures the expression level of each sample from the perspective of the overall dispersion of expression quantity.

**Figure S2. The distribution of differentially expressed lncRNA and mRNA sequences in chromosomes.** The outer ring is the chromosome of the reference genome of the species, the middle ring is the distribution of differentially expressed mRNA on the chromosome, and the inner ring is the distribution of differentially expressed lncRNA on the chromosome. In the ring, red represents up-regulated genes, green represents down-regulated genes, yellow represents up-regulated lncRNA, and blue represents down-regulated lncRNA.


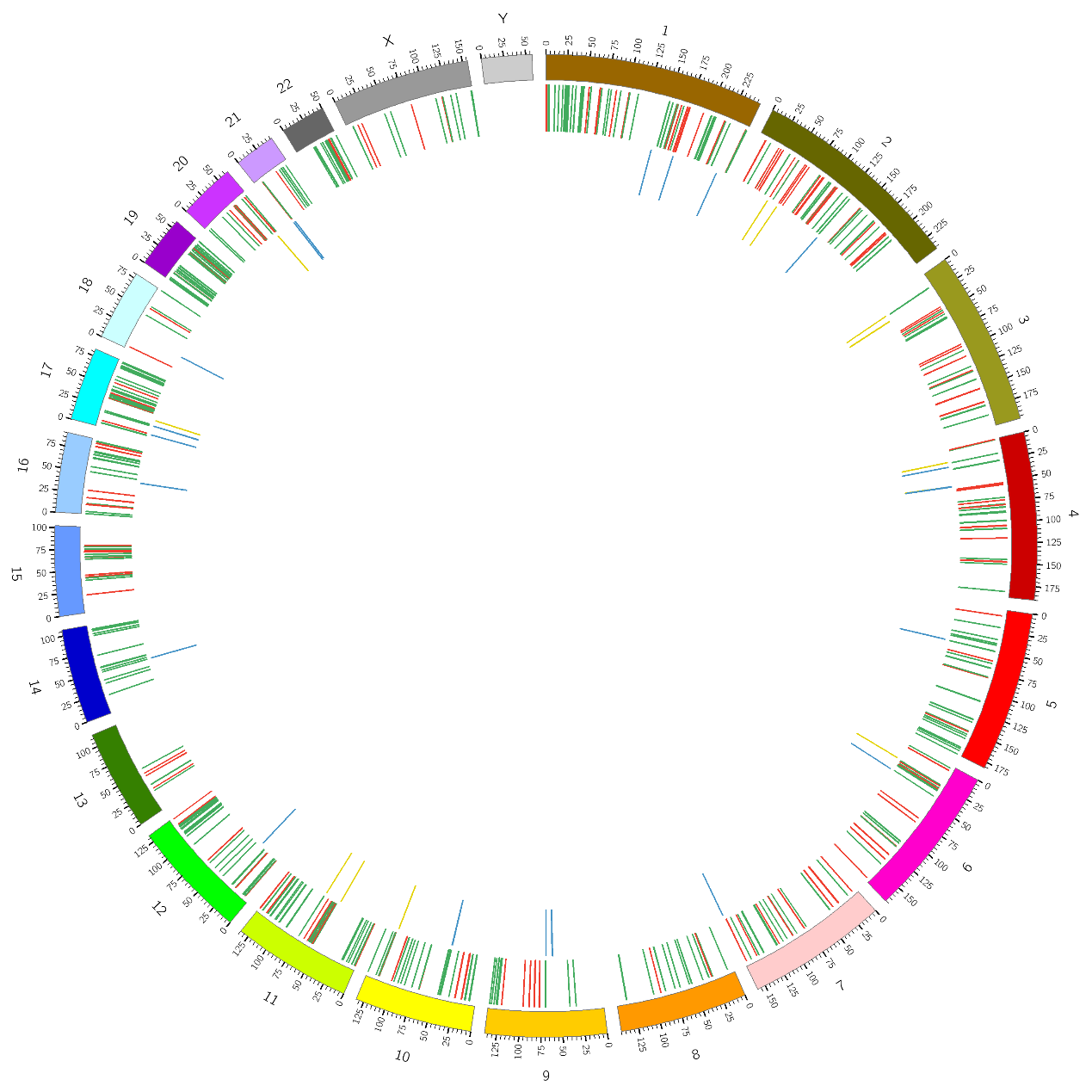


**Figure S3. GO classification statistics of differentially expressed genes among samples.** The abscissa is the GO classification, the left ordinate is the percentage of the number of genes, and the right ordinate is the number of genes.


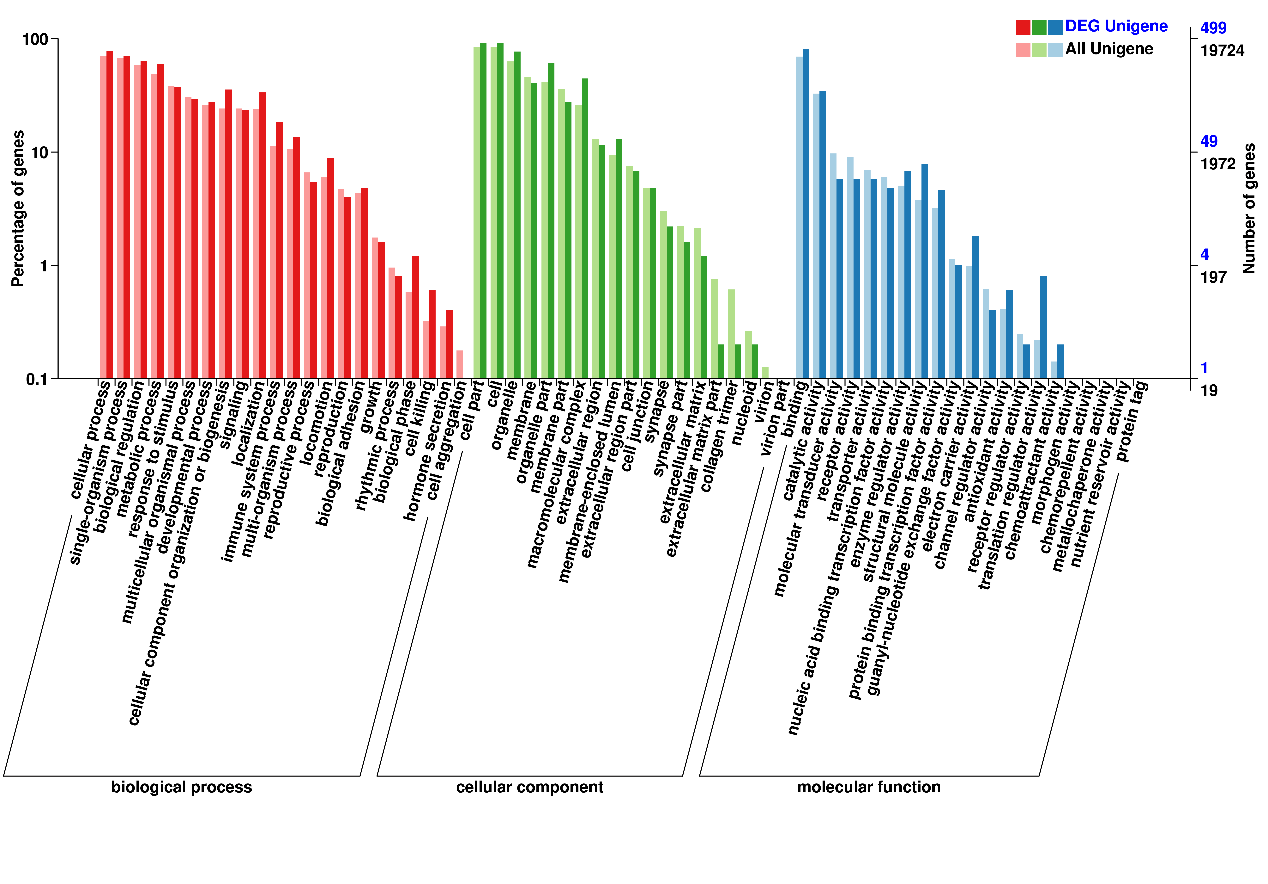


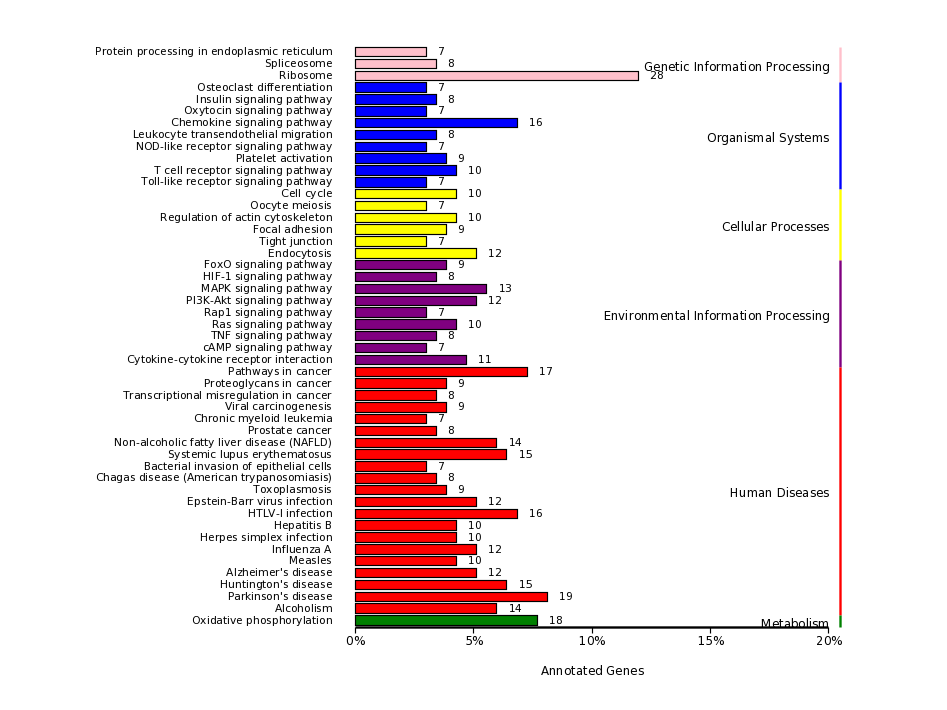
**Figure S4. The annotation results of differentially expressed KEGG genes were classified according to the pathway types in KEGG.** The vertical coordinate is the name of KEGG metabolic pathway, and the horizontal coordinate is the number of genes annotated to this pathway and their proportion in the total number of genes annotated.


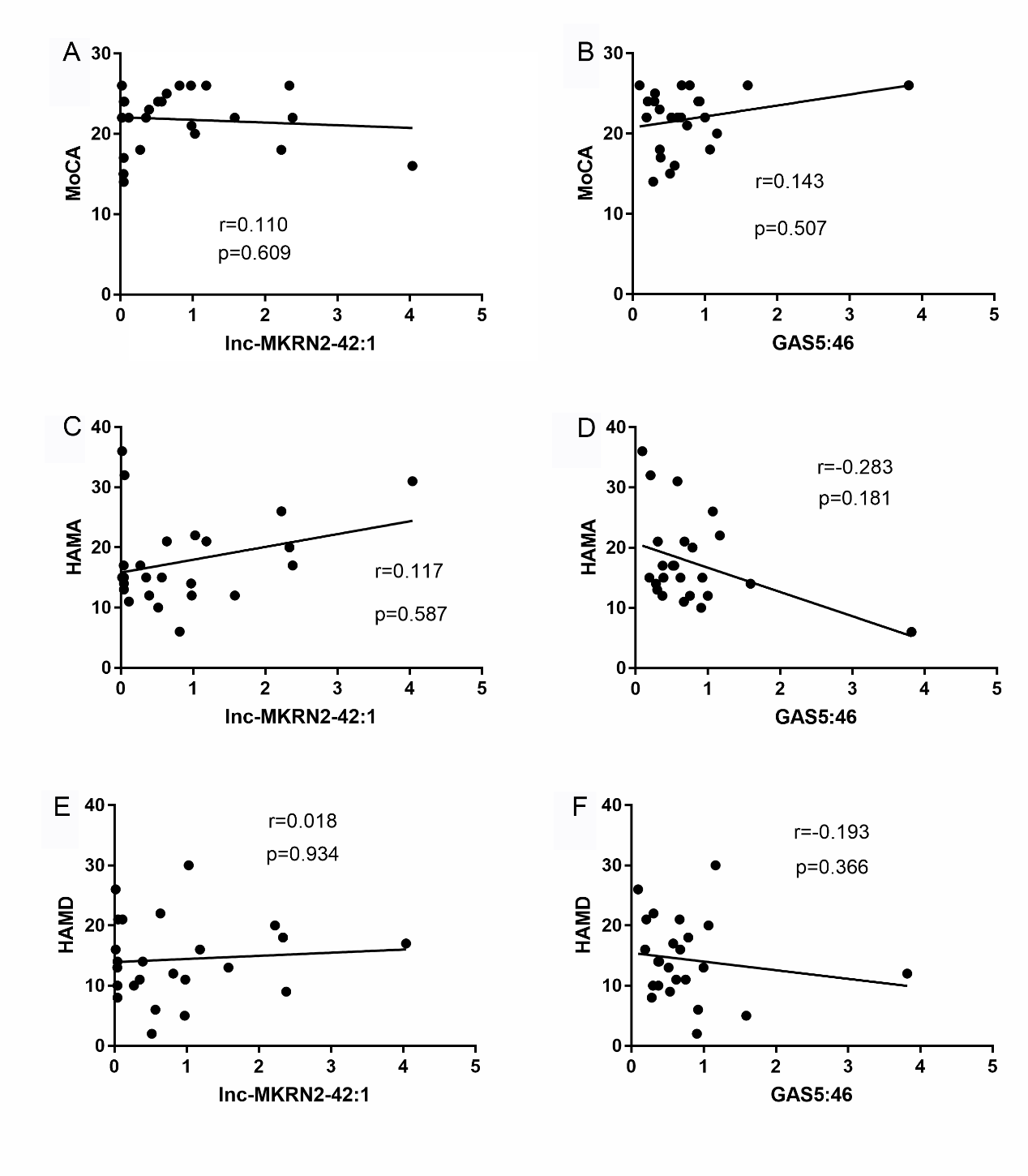
**Figure S5. Correlation analysis of lncRNA expression level and clinical characteristics.** Correlations according to spearman coefficient between the related expression level of lncRNAs and clinical charactors in PD patients. (n = 24). MoCA, Montreal Cognitive Assessment; HAMD, Hamilton Depression Scale; HAMA, Hamilton Anxiety Scale.

**Figure S6. The corresponding number of four diffirent LncRNA.** lncRNAs were screened using CPC/CNCI/Pfam, which has the power to distinguish the protein-coding genes from the non-coding genes. In addition to the different types of lncRNAs, lincRNA, intronic lncRNA, and anti-sense lncRNAs were selected using Cuffcompare.


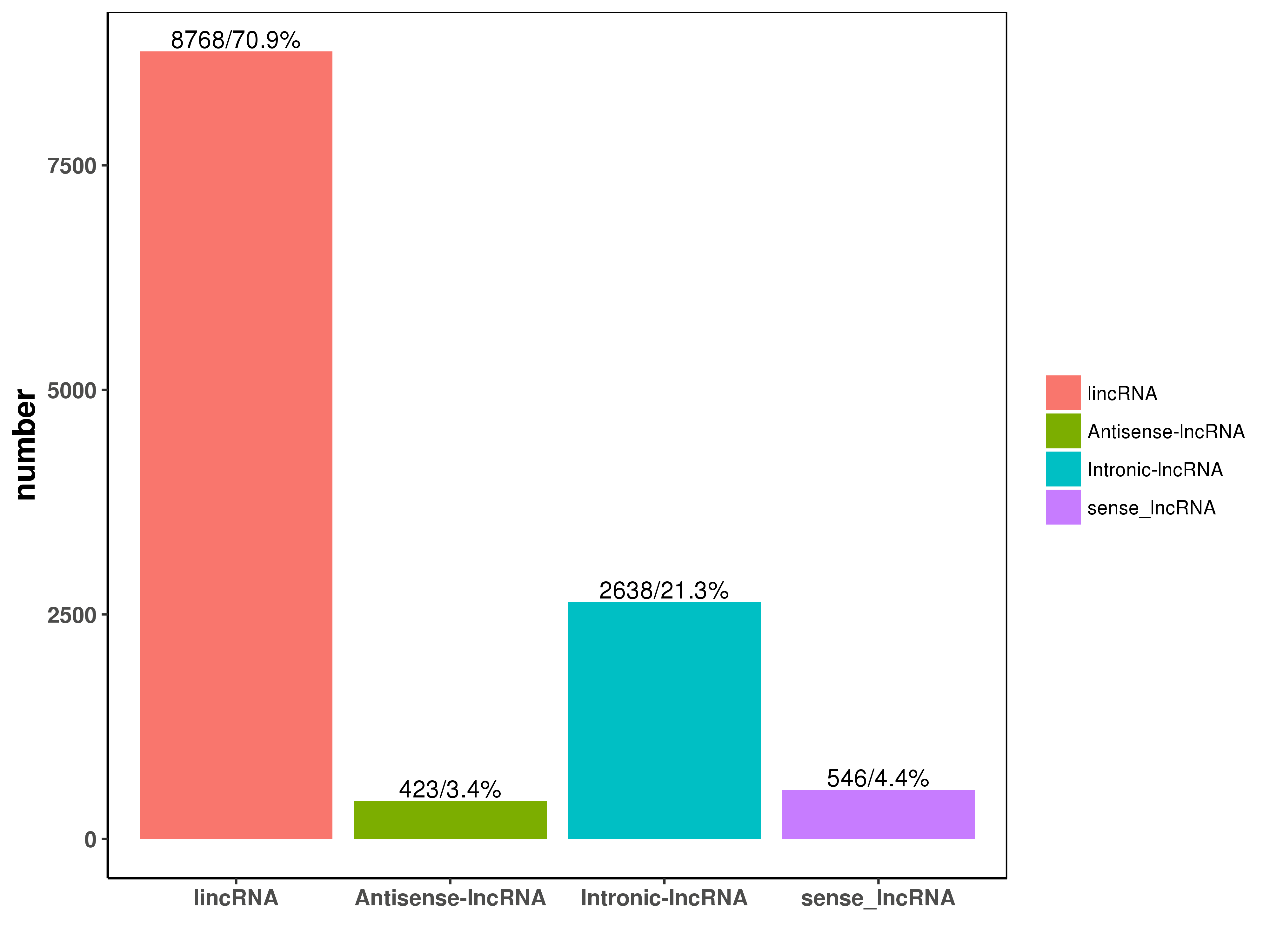


**Table S1.** **Up- and down-regulated lncRNAs revealed by sequencing. log2FC: log2 Fold Change.**

| #ID | P value | log2FC | regulated |
| --- | --- | --- | --- |
| MSTRG.336210.1 | 0.006354 | -7.99115 | down |
| lnc-MKRN2-42:1 | 0.006354 | -5.30754 | down |
| lnc-ZFAND5-29:1 | 0.020362 | -3.88016 | down |
| MSTRG.36313.1 | 0.004041 | -3.82246 | down |
| MSTRG.28948.3 | 0.040029 | -3.75945 | down |
| MSTRG.296817.4 | 0.026224 | -3.51459 | down |
| MSTRG.103195.10 | 0.018661 | -3.03459 | down |
| GAS5:46 | 0.049973 | -2.78099 | down |
| MSTRG.132814.8 | 0.020881 | -2.69376 | down |
| lnc-N6AMT1-156:1 | 0.01388 | -2.52345 | down |
| lnc-CUL2-56:1 | 0.029125 | -2.35318 | down |
| lnc-NPY4R2-105:1 | 0.006993 | -2.14101 | down |
| MSTRG.362284.4 | 0.040029 | -1.99109 | down |
| lnc-FCGR1B-16:1 | 0.006993 | -1.83443 | down |
| MSTRG.202295.1 | 0.037879 | -1.70083 | down |
| MSTRG.202898.1 | 0.029662 | -1.68712 | down |
| lnc-GLP2R-144:1 | 0.040692 | -1.6625 | down |
| MSTRG.134415.2 | 0.017483 | -1.57523 | down |
| MSTRG.246606.1 | 0.040692 | -1.55113 | down |
| MSTRG.16384.25 | 0.017483 | -1.52767 | down |
| MSTRG.126052.1 | 0.037879 | -1.19565 | down |
| MSTRG.16384.29 | 0.037879 | -1.08508 | down |
| lnc-GPT-59:1 | 0.037879 | -1.08218 | down |
| MSTRG.144437.1 | 0.017483 | -0.71719 | down |
| MSTRG.16383.2 | 0.037879 | 1.648264 | up |
| MSTRG.294578.11 | 0.039266 | 2.293659 | up |
| lnc-FCGR1B-16:1 | 0.010518 | 2.909011 | up |
| MSTRG.372578.1 | 0.026224 | 2.973041 | up |
| MSTRG.58786.4 | 0.020362 | 3.02425 | up |
| MSTRG.55826.1 | 0.026224 | 3.193948 | up |
| lnc-NSMCE4A-84:4 | 0.009528 | 3.388173 | up |
| MSTRG.201403.1 | 0.020362 | 3.520246 | up |
| MSTRG.214866.5 | 0.019945 | 3.576613 | up |
| MSTRG.135068.4 | 0.045215 | 3.823103 | up |
| MSTRG.246522.65 | 0.040692 | 3.859273 | up |
| MSTRG.215953.1 | 0.010264 | 4.303947 | up |
| MSTRG.167268.5 | 0.014047 | 5.049997 | up |
| MSTRG.242001.1 | 0.029125 | 5.550372 | up |
| MSTRG.169261.1 | 0.009528 | 8.096841 | up |

**Table S2.** **Most differentially- expressed genes revealed by sequencing analysis.** The table only lists the top 62 up- and downregulated genes based on the fold change values. log2FC: log2 Fold Change.

| #ID | P-value | log2FC | regulated |
| --- | --- | --- | --- |
| NME4 | 0.002222 | -7.10412 | down |
| CD3D | 0.009504 | -6.18994 | down |
| ECSCR | 0.006354 | -5.5444 | down |
| MICALCL | 0.040686 | -5.24397 | down |
| ECHO1805-A20_newGene_176942 | 0.040686 | -4.97297 | down |
| ZNF708 | 0.00475 | -4.79733 | down |
| RABGGTB | 0.00193 | -4.49911 | down |
| ZNF562 | 0.040692 | -4.47568 | down |
| GORASP2 | 0.002069 | -4.28171 | down |
| FBXO33 | 0.009764 | -4.26973 | down |
| PPIL2 | 0.01388 | -4.24323 | down |
| HDHD1 | 0.013031 | -4.22069 | down |
| EPB41L4A | 0.007226 | -4.19709 | down |
| FIP1L1, LNX1 | 0.040029 | -4.17235 | down |
| SLFN14 | 0.014094 | -4.04257 | down |
| TMEM69 | 0.049973 | -4.0018 | down |
| MT-ND4L, MT-ND4 | 0.001166 | 4.068098 | up |
| ECHO1805-A20_newGene_91525 | 0.017483 | 4.084404 | up |
| ECHO1805-A20_newGene_157396 | 0.006662 | 4.088917 | up |
| SV2C | 0.017483 | 4.097273 | up |
| C2orf88, HIBCH | 0.011072 | 4.182931 | up |
| LMBR1 | 0.002331 | 4.188349 | up |
| TSC22D1 | 0.006993 | 4.227151 | up |
| PF4 | 0.000583 | 4.253368 | up |
| CLU | 0.001166 | 4.261154 | up |
| PPBP | 0.000583 | 4.266546 | up |
| MEIS1 | 0.001166 | 4.357748 | up |
| JAM3 | 0.037879 | 4.38809 | up |
| B2M | 0.000583 | 4.392245 | up |
| LEPR, LEPROT | 0.037879 | 4.401716 | up |
| BEND2 | 0.033019 | 4.465267 | up |
| RP5-857K21.4, hsa-mir-6723, RP5-857K21.7 | 0.047401 | 4.559586 | up |
| CD9 | 0.004041 | 4.585313 | up |
| C6orf25 | 0.020881 | 4.602514 | up |
| ECHO1805-A20_newGene_99565 | 0.010518 | 4.658326 | up |
| CCDC101 | 0.026313 | 4.660753 | up |
| TRMT13 | 0.010518 | 4.663432 | up |
| ECHO1805-A20_newGene_46895 | 0.001166 | 4.666575 | up |
| NPTN | 0.002141 | 4.67571 | up |
| ECHO1805-A20_newGene_230122 | 0.00193 | 4.696204 | up |
| MMD | 0.004079 | 4.823392 | up |
| SEMA4D | 0.026224 | 4.837227 | up |
| RP5-1043L13.1 | 0.006354 | 4.944936 | up |
| PROS1 | 0.026313 | 5.124479 | up |
| RP11-33B1.1, PDE5A | 0.011072 | 5.223795 | up |
| LGALSL | 0.02806 | 5.427621 | up |
| PRKAR2B, CTA-360L10.1 | 0.002331 | 5.442838 | up |
| DSE, RP3-486I3.7, RP3-486I3.4, TSPYL1, RP1-93H18.1 | 0.011072 | 5.46432 | up |
| MAP3K7CL | 0.000583 | 5.469587 | up |
| MT-ND1 | 0.037879 | 5.64258 | up |
| RGS18 | 0.000583 | 5.748411 | up |
| ECHO1805-A20_newGene_43137 | 0.013031 | 5.776807 | up |
| MED12L | 0.000583 | 5.786592 | up |
| MORC1 | 0.020881 | 6.002837 | up |
| DDX11L1 | 0.00326 | 6.135823 | up |
| PGRMC1 | 0.013031 | 6.306819 | up |
| PF4V1 | 0.001711 | 6.402415 | up |
| PTGS1, RP11-498E2.9, AL162424.1 | 0.010518 | 6.628186 | up |
| CXCL5 | 0.033019 | 6.676608 | up |
| ENKUR | 0.01388 | 7.39794 | up |
| ECHO1805-A20_newGene_44640 | 0.006354 | 9.525665 | up |
| ACRBP | 0.009504 | 9.808702 | up |
